# Giant change of MoS_2_ optical properties along amorphous–crystalline transition: broadband spectroscopic study including the NIR therapeutic window†

**DOI:** 10.1039/d3na00111c

**Published:** 2023-04-18

**Authors:** Jan Mistrik, Milos Krbal, Vit Prokop, Jan Prikryl

**Affiliations:** a Center of Materials and Nanotechnologies, Faculty of Chemical-technology, University of Pardubice Studentska 95 53210 Pardubice Czech Republic jan.mistrik@upce.cz +420 466 037 409; b Institute of Applied Physics and Mathematics, Faculty of Chemical-technology, University of Pardubice Studentska 95 53210 Pardubice Czech Republic

## Abstract

This work deals with an ellipsometric study of magnetron sputtered thin MoS_2_ films. The evolution of the UV-VIS-NIR optical properties of as-deposited and subsequently annealed films is thoughtfully investigated, covering amorphous, amorphous relaxed, partially crystallized, and polycrystallized MoS_2_ films. The transition from the mixed 1T′@2H local order in the amorphous phase toward the long-range 2H order in the polycrystalline phase is systematically correlated with film optical properties. The early stage of a few-layer 2H ordering toward the 2H bulk-like polycrystalline structure during annealing is evidenced through the energy shift of MoS_2_ prominent excitonic peaks. A considerable change in optical response between metallic (amorphous) and semiconducting (polycrystalline) MoS_2_ phases is reported and presented in terms of dielectric permittivity and normal reflectance NIR-VIS-UV spectra. Results of light–heat conversion in the NIR therapeutic window show so far uncovered potential of amorphous MoS_2_ as an agent for photothermal therapy. Spectroscopic ellipsometry provided sensitive characterization disclosing essential results complementary to other characterization tools. The benefit of these results is expected to be employed in fundamental and application-motivated research, for example, in the field of phase change materials, photothermal cancer therapy, and magneto-optical study of magnetic ordering in metal transition dichalcogenides, among others.

## Abstract 2

The annealing of MoS_2_ films shows a transition from the mixed 1T′@2H local order (amorphous) toward the long-range 2H order (polycrystalline). So far uncovered potential of amorphous MoS_2_ as an agent for photothermal therapy is evidenced.

## Graphical abstract

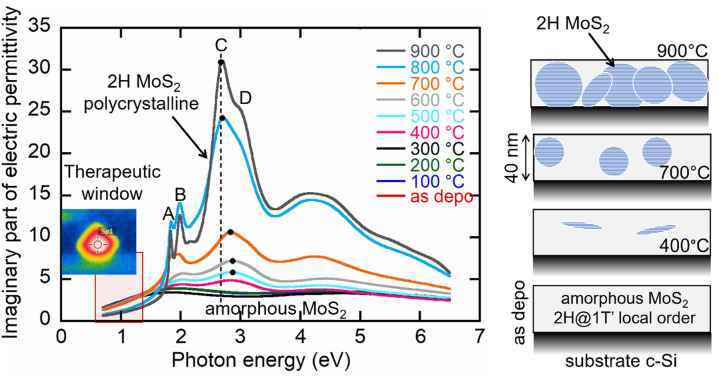

## 1 Introduction

MoS_2_ is an intensively studied material in its both bulk-like and reduced dimensionality forms, offering a plethora of new promising applications due to its rich and tunable structural properties.^1–3^ Owing to its polymorphism, crystalline MoS_2_ offers various phases, which contain Mo and S atoms arranged in different geometries, for example, octahedral (1T) or trigonal prismatic (2H). Nevertheless, all the crystalline phases present a layered structure built by parallel slabs (three atomic layers) of covalently bonded S–Mo–S atoms. The slabs are weakly bonded by van der Waals interactions. The discrepancy in the strength and nature of in-plane and out-of-plain bonding causes strong optical anisotropy (large birefringence phenomena) that can be employed, for example, for a light manipulation in photonic chips.^4^ Different bonding and symmetry of Mo and S atoms induce different physical properties and the manifestation of 1T and 2H phases. 2H MoS_2_ is semiconducting, whereas 1T is metallic.^5,6^ Moreover, the 2H phase is nonmagnetic, but the 1T phase shows ferromagnetic behavior.^7^ 2H MoS_2_ presents an indirect to direct band gap transition when reducing thickness to the ultrathin limit.^8,9^ The photoluminescence of the 2H MoS_2_ monolayer is employed in optoelectronic applications.^1^ In its 2D form MoS_2_ shows fundamentally interesting excitonic magneto-optical activity induced by the spin proximity effect.^10^ Although the metallic 1T phase has limitations for optoelectronic applications, it becomes attractive as a catalyst for hydrogen evolution, photocatalysts, or supercapacitor electrode materials.^11,12^ Owing to optical absorption in NIR, the metallic 1T phase was proven to be an efficient agent for photothermal therapy in the near-infrared therapeutic window, as well.^13–15^ Unfortunately, this phase is thermodynamically unstable and easily converts to 2H. Nevertheless, it was recently reported that the stable MoS_2_ amorphous phase, with a mixture of 1T and 2H local atomic arrangement, reveals a similar bond structure to that in the 1T phase.^16^ This predicts promising applications of the less studied MoS_2_ amorphous phase in various fields, for example, high activity of amorphous MoS_2_ in the hydrogen evolution reaction^16^ or cancer treatment by photothermal therapy. The knowledge of the optical properties of the amorphous phase and their evolution due to bond rearrangement toward the 2H phase by thermal annealing is therefore crucial for both theoretical and applied research.

On the other hand, owing to the attractiveness of MoS_2_ properties, up-scalable methods for the fabrication of MoS_2_ in its various phases and dimensions are constantly searched. One relatively simple method of 2H MoS_2_ preparation is magnetron sputtering of its amorphous phase with subsequent annealing. It is of fundamental and application interest the exact knowledge of phase change in this particular case. Monitoring the evolution of the optical properties of annealed films not only provides a characterization tool for this transformation but also offers information on their large variation during the phase change.

This work focuses on the ellipsometric study of magnetron sputtered thin MoS_2_ films. The concern is devoted to the evolution of the optical properties of as-deposited and subsequently annealed films. This corresponds with amorphous, amorphous relaxed, partially crystallized, and polycrystallized MoS_2_ films. In other words, the transition from the amorphous mixed phase of tetragonal distorted (1T′) and the trigonal prismatic (2H) local order (denoted here as 1T′@2H) toward the long-range 2H order in the polycrystalline phase is systematically studied through film optical property modification. Evidence of a few-layer 2H ordering toward a bulk-like polycrystalline structure during annealing is discussed through the energy shift of MoS_2_ prominent excitonic peaks. A huge change in optical response between metallic (amorphous) and semiconducting (polycrystalline) MoS_2_ phases is reported and presented in terms of determined dielectric permittivity and normal reflectance NIR-VIS-UV spectra. The optical properties of the amorphous MoS_2_ phase make an original contribution to the field. Additionally, results of light–heat conversion in the NIR therapeutic window show so far uncovered potential of amorphous MoS_2_ as an agent for photothermal therapy. Ellipsometry is presented as a rather sensitive characterization tool providing both supporting and complementary results to other material characterization techniques. The value of these results is expected to be found in fundamental and application-motivated research, for example, in phase change materials,^17^ photothermal therapy, and magneto-optical characterization of MoS_2_ magnetism, among others.

## 2 Experimental

Amorphous MoS_2_ films were sputter deposited onto naturally oxidized c-Si wafers. Their backsides were intentionally grounded to prevent backside reflections in the NIR range. Subsequent annealing in sealed quartz ampules at a residual pressure of 10^−3^ Pa to prevent oxidation was performed for selected temperatures up to 900 °C. A furnace with a heating rate of 2 °C min^−1^ was used. Samples were kept at the given temperature for one hour and then naturally cooled down to room temperature. Complementary characterization by XPS, XAS, sheet resistance, and XRD was previously reported in ref. 6 and 18.

Ellipsometric spectra were recorded by using a rotating analyzer VASE ellipsometer (Woollam, Co. Ltd) in the spectral range from 0.7 to 6.5 eV at incidence angles 50°, 60°, and 70°. Nearly normal (angle of incidence 18°, p-incident polarization) reflectance measurements were carried out by using the same instrument in a one-beam configuration. Normal incidence transmittance measurements performed on MoS_2_ films deposited under the same conditions on transparent fused silica substrates were scanned by using a JASCO V-570 spectrophotometer. Besides ellipsometric parameters *Ψ* and Δ (ratio of *r*_p_ and *r*_s_ coefficients and their mutual shift), the degree of depolarization was monitored as well. Ellipsometry is a phase sensitive technique and, when employed in variable angle spectroscopic configuration and further coupled with spectrophotometry (reflectance and transmittance), provides precise and accurate results. Ellipsometry, an indirect characterization tool, requires the construction of a sample model for data interpretation. In our case, a semi-infinitive c-Si substrate, SiO_2_ overlayer (native oxide), and MoS_2_ film were employed in reflection configuration, whereas a fused silica substrate and MoS_2_ film were employed for transmittance measurements. Surface roughness that was considered by Bruggeman effective medium approximation (with 50% of voids) can simultaneously model slight surface oxidization as well. Sputtering yields uniform films that are corroborated by low depolarization values. Modeling does not show any index gradient. Therefore MoS_2_ is modeled as uniform homogeneous single layers. The optical constants of c-Si and SiO_2_ native oxide were taken from the software database WVASE32.

Light–heat conversion in the NIR therapeutic window was examined by exposure of selected films (as deposited and 900 °C annealed) to supercontinuum radiation (WhiteLase Micro, NKT Photonics). A longpass optical filter was used to cut wavelengths lower than 1000 nm, and therefore a collimated 4 mm diameter polychromatic (1000–2200 nm) beam was directed to the film surface under the angle of incidence of 45 deg. The total spectral power of the incident beam was 340 mW. The film temperature increase was monitored by using an FLIR i7 infrared camera (FLIR System, Inc.). The emissivity of film surfaces was set to 0.6. NIR transparent fused silica substrates were used to avoid light absorption and subsequent heat generation.

## 3 Model dielectric function of MoS_2_

The model dielectric function (MDF) of MoS_2_ in the NIR-VIS-UV range is usually constructed by a sum of oscillators of various types (for example, Gauss, Lorentz, or Tauc–Lorentz), the number of which depends on the available spectral range and spectral resolution.^19–22^ The appropriateness of oscillator type selection is discussed in ref. 19. Nevertheless, other mathematical functions specifically designed for electronic transitions in the vicinity of MoS_2_ band structure critical points and rich excitonic features were employed as well.^21,23^ Our choice is the sum of Lorentz oscillators due to its high appearance in the literature that facilitates a straightforward comparison of the obtained results. As will be discussed later in the text, the number of oscillators depends on the annealing temperature starting from 3 (as-deposited films) and reaching the number of 9 (900 °C annealed polycrystalline films). Hence, the model dielectric function gets the form1
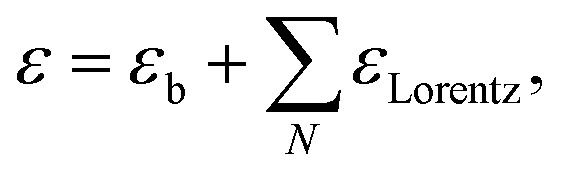
where the contributions to the real part of the dielectric function from high photon energy transitions are accounted for by a pole *ε*_b_. The Lorentz oscillator is expressed as2
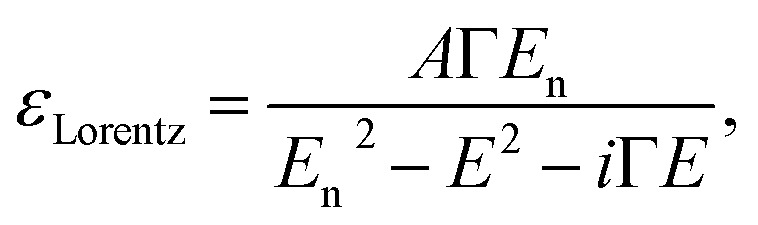
where *A* stands for the oscillator strength, *E*_n_ the transition energy, *Γ* the broadening, and *E* the energy of interacting photons. Even if MoS_2_ is strongly optically anisotropic in its crystalline form (uniaxial anisotropy), we consider our films to be optically isotropic due to their predominately amorphous and poly-crystalline nature. A particular case of textured films annealed with the highest temperature of 900 °C is discussed later.

## 4 Results and discussion

**Fig. 1:**
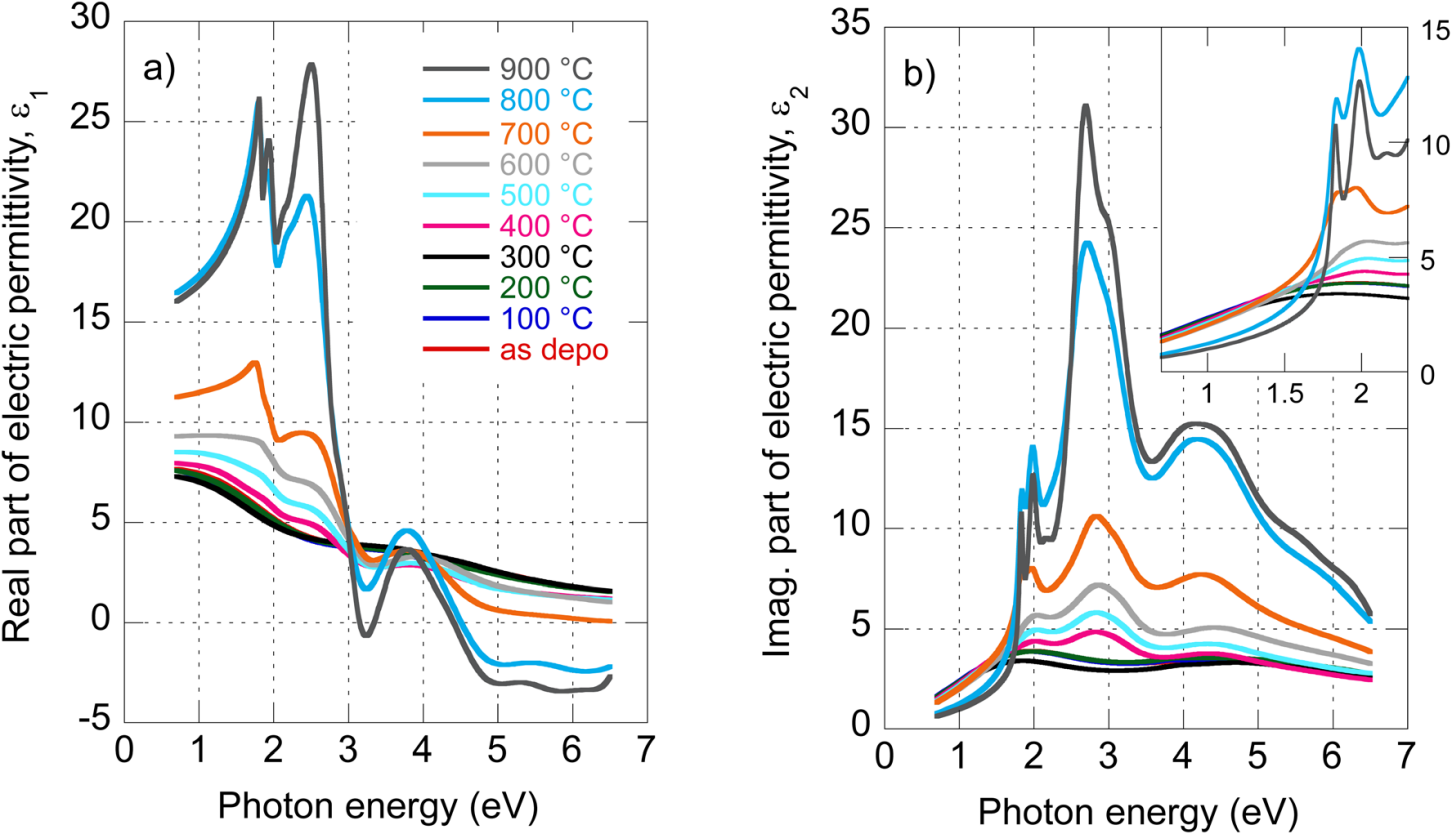
Determined real part (a) and imaginary part (b) of MoS_2_ electric permittivity spectra as a function of annealing temperature.

The evolution of optical constants, displayed in the form of real and imaginary parts of MoS_2_ dielectric permittivity, *versus* annealing temperature is presented in Fig. 1 (for the wavelength dependence of the refractive index and extinction coefficient, refer to ESI, Fig. S3).† The following sections are devoted to interpreting these changes in terms of MoS_2_ transformation from its amorphous (as-deposited) phases to the 2H polycrystalline phase (films annealed with the highest temperatures 800 °C and 900 °C).

### 4.1 2H polycrystalline phase (900 °C annealed film)

We start our discussion with the polycrystalline film annealed at the highest temperature of 900 °C. Regarding our recent paper,^18^ this film shows a nearly textured polycrystalline 2H phase with a negligible contribution of other phases (supported by XPS spectra deconvolution). The films are semiconducting with a sheet resistance of about 10^7^ Ω cm^−2^ that is expected for the 2H MoS_2_ phase.^5^

The optical properties of 2H MoS_2_ were extensively studied in its bulk and (ultra)thin film forms (refer, for example, to ref. 4, 19 and 23–25). The strong light–matter coupling of 2H MoS_2_ in the VIS range includes prominent features of exciton contributions generally labeled A, B, C, and D. It has been reported that the spectral positions of these excitons are, in the ultrathin limit (dimensions less than about 4 nm), more or less thickness dependent due to the quantum confinement. Additionally, the quantum confinement induces 2H MoS_2_ band structure modifications that transform its band gap from the indirect (bulk) to the direct (monolayer) form. This effect was initially reported in photoluminescence studies.^8^ In our case, ellipsometry measurements revealed a film thickness of about 20 nm, and therefore, no significant quantum confinement or band gap modification is expected in our polycrystalline 2H MoS_2_ films.

**Fig. 2:**
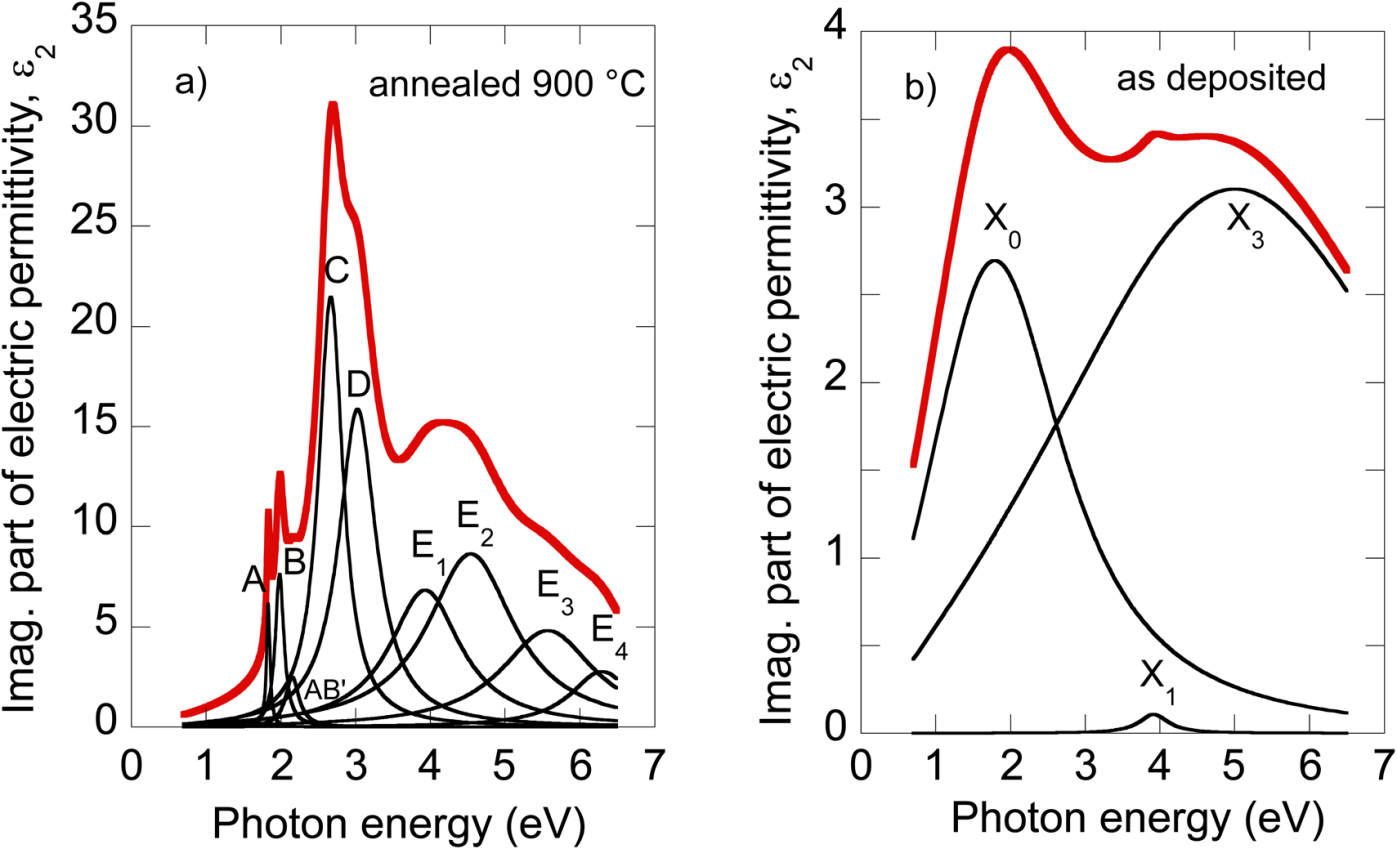
Deconvolution of the MoS_2_ optical constants of the 900 °C annealed film (a) and as-deposited film (b).

The electric permittivity of the film was parameterized by 9 Lorentz oscillators accounting for the excitons and critical points of the Brillouin zone. The individual contributions are presented in Fig. 2a in the form of its imaginary part. In the VIS range, the excitonic features A, B, C, and D dominate over other contributions and are clearly identified. The origins and assignments of these transitions are discussed in the literature (see, for example, ref. 20, 21 and references therein). The A and B peaks are assigned to the transition from the spin–orbit split valence band to the lowest conduction band at the K and K′ points, whereas the C and D excitons are predominantly associated with the transition in the part of the Brillouin zone (BZ) between K and Gamma points with parallel valence and conduction bands.^8,26^ The positions of excitonic peaks that we have determined are mentioned in Table 1. Their respective values are *E*_A_ = 1.83 eV, *E*_B_ = 1.98 eV, *E*_C_ = 2.67 eV, and *E*_D_ = 3.04 eV, which are representative values for bulk like MoS_2_.^21,23,27,28^

Adjusted parameters, electronic transitions, film thickness, and surface roughness, determined from spectroscopic ellipsometry for as-deposited and annealed films. MSE parameter stands for mean square error and evaluates fit quality. Energies are provided in eV, thickness in nm, and temperatures in °C

**Table:** 

Temp.	Num.	*Y*_0_	A	B	AB′	C	D	*E*_1_	*E*_2_	*E*_3_	*E*_4_	SR	*t*	MSE
900	9		1.83	1.98	2.15	2.67	3.04	3.97	4.59	5.62	6.31	1.3	22.7	6
800	8		1.83	1.98	2.22	2.67	3.04	4.03	4.59	5.92		0.0	21.4	3
700	8	2.68	1.83	1.97		2.83	3.15		4.34	5.39	6.47	0.0	32.5	2
600	8	2.39	1.87	2.00		2.87	3.19		4.50	5.63	6.74	0.0	57.1	2
500	8	2.09	1.88	2.02		2.87	3.18		4.51	5.70	7.11	0.0	62.1	1
400	8	1.61/2.41		2.01		2.84	3.16		4.48	5.90	6.91	1.3	65.8	2

**Table:** 

		*X*_0_						*X*_1_		*X*_2_	*X*_3_	SR	*t*	MSE
300	4	1.99						3.95		4.78	6.09	0.3	56.2	2
200	4	2.16						3.86		5.30	6.70	0.0	54.6	2
100	4	2.17						3.79		5.06	6.90	0.0	57.2	2
As-deposited	3	2.12						3.92			5.89	0.8	57.2	2

The oscillator strength of A, B, and C excitons reported in the literature spans over a broad range (see, for example, ref. 29 and references therein). It should be noted here that for correct exciton amplitude determination, the transmittance spectra should be added and treated in parallel with ellipsometry and reflectance spectra. This approach minimizes the correlation between film thickness and MoS_2_ extinction in the film semitransparent spectral region – the issue often encountered for ellipsometry treatment of thin absorbing films. In our case, transmittance measurements carried out on MoS_2_ films deposited and annealed under the same conditions on transparent fused silica substrates were included in data (ellipsometry and reflectance) treatment, and hence, controlling the final fit and reliability of the values of adjusted parameters. More information about transmittance measurements is reported in the ESI (see Fig. S1).†

It is worth noting that excited states of A and B excitons were also detected and labeled (AB)′ oscillator in Fig. 2a. Hence, well-developed exciton features A, B, C, and D together with the presence of excited states (AB)′ prove the high quality of the 2H MoS_2_ film.

Other oscillators identified in UV (3.5–6 eV), labeled here *E*_1_–*E*_4_ (*cf.*Table 1), are generally interpreted as electronic transitions between the valence band and conduction and exited bands of BZ in its high symmetry points.^20,21^ A different number of oscillators (for example, two^20,22^ or four^21,30^) were used in the literature to parameterize the 2H MoS_2_ electric permittivity in this spectral range. The assignment of these transition energies depends on spectra deconvolution and is, in general, difficult due to the complex nature of the excited bands of MoS_2_ BZ.

It is known that monocrystalline MoS_2_ presents strong optical anisotropy,^4^ and therefore texturing or preferential crystal orientation can be an issue for ellipsometry data treatment. Preferential (002) orientation of the 900 °C annealed film was reported in our previous XRD study.^18^ In this case, the uniaxial optical axis is perpendicular to the sample surface. Therefore, only effective optical constants are determined from oblique light incidence ellipsometry measurements (in-plane and out-of-plane contributions cannot be separated). Nevertheless, due to the high MoS_2_ refractive index value, light refracted to the film propagates nearly perpendicularly to the surface and, therefore, senses mainly in-plane permittivity contribution. Out-of-plane permittivity represented by weak vdW bonds does not show any spectral structures in VIS and can be parameterized by the simple Cauchy dispersion relation as reported in ref. 4.

Defect-free bulk 2H MoS_2_ monocrystals are transparent in NIR for photon energies lower than the onset of A-exciton absorption.^24^ Nevertheless, our polycrystalline film shows a moderate absorption tail due to structural imperfections or defects. This was similarly detected and discussed, for example, by Singh *et al.*, who have recently disclosed the importance of the near-infrared optical properties of transition metal disulfides, including MoS_2_, searching new alternative phase change materials.^17^

### 4.2 Amorphous phase (as-deposited film)

Our recent X-ray study proved the amorphous phase in as-deposited films. Moreover, XPS characterization showed bonds with local symmetry of a 2H@1T′ mixed phase,^18^ quantitatively estimated to be around 50% each with only a slight contribution of MoO_*x*_ on the film surface. The sheet resistance of as-deposited films (about 10^3^ Ω cm^−2^) was four orders lower with respect to the polycrystalline 2H phase mainly due to the Mo–Mo bond network, which is not present in the 2H phase but built *via* partial 1T′ local ordering in the 2H@1T′ mixed phase.^18^ The change in MoS_2_ electrical transport properties from the tetragonal 1T (or distorted 1T′) metallic phase containing Mo–Mo bonds to the hexagonal 2H semiconducting phase is well known (see, for example, ref. 5).

To our knowledge, the optical properties of amorphous MoS_2_ films have not been systematically investigated. We can report only absorbance measurements on amorphous MoS_2_ films^31^ or nanoparticle suspensions.^32^ In contrast to the (poly)crystalline 2H phase, no excitonic features are observed in the amorphous phase, as is evident from Fig. 2b, where we present the imaginary part of as-deposited film electric permittivity. The significantly lower absorption (lower light–matter coupling) in the amorphous phase in the VIS is also worth noting. In contrast to the NIR, the amorphous phase shows enhanced extinction over the crystalline phase (*cf.* also Fig. 1b). This could be an unexpected result considering that sulfur-based amorphous chalcogenides such as As_2_S_3_ and related materials with the so-called valence alternation pairs are highly transparent in the NIR.^33^ Nevertheless, amorphous MoS_2_ does not show this optical behavior. This is probably due to the absence of valence alternation pairs (VAPs) in the amorphous phase and the existence of homopolar metallic Mo–Mo bonds.^18^ Accordingly, the effect of band gap shrinking and development of an absorption tail in the NIR was recently reported for an amorphous thin film along the As_40_S_60_–MoS_3_ tie-line.^34^

Owing to less featured spectral dependence of amorphous MoS_2_ electric permittivity (compared to the crystalline one), its deconvolution consists of only 3 Lorentz oscillators as indicated in Fig. 2b and Table 1. One contribution labeled *X*_0_ is located at 2.1 eV, and the other two labeled *X*_1_ and *X*_3_ at 3.9 and 5.9 eV, respectively. Considering the metallic nature of the amorphous phase with the contribution of the homopolar Mo–Mo atomic network, we compare the identified electronic transitions with those reported for metallic molybdenum localized at 1.7, 2.3, and 2.4 eV.^35^ Accordingly, we suggest that the *X*_0_ transition at 2.1 eV relates mainly (but not solely) to the homopolar Mo–Mo metallic bonds. As will be shown later, this transition remains present for films annealed up to 600 °C but disappears for highly annealed (700–900 °C) films with a dominantly 2H semiconductor nature.

The other electronic transitions *X*_1_ and *X*_3_ occur in the spectral range where 2H polycrystalline MoS_2_ features several, *E*_1_–*E*_4_, valence to conduction, and higher band transitions (*cf.*Table 1). However, their precise assignment is difficult due to the mixed contribution of 1T′ and 2H locally coordinated S–S and Mo–S bonds. Nevertheless, it is interesting to point out the close position of *X*_1_ and *E*_1_, and *X*_3_ and *E*_4_ transitions disclosed in the mixed 1T′@2H amorphous and 2H polycrystalline phases.

Considering the huge difference between the optical properties of 2H polycrystalline and amorphous MoS_2_, photonic devices based on order–disorder switching are, in principle, realizable. On the other hand, switching energy (annealing up to 900 °C) is unacceptably high. To overcome this problem designing thermodynamically stable alloys based on transition metal disulfides, including MoS_2_, is an alternative being explored.^3,17^ Other routes toward amorphous-to-crystalline phase transformation in MoS_2_ as ion or electron beam irradiation are investigated as well,^36,37^ and knowledge of the optical properties of the amorphous phase is beneficial for its characterization or metasurface based photonic applications.

Another benefit from detected enhanced absorption of amorphous MoS_2_ in the NIR that covers the therapeutic window would be its application in photo-thermal therapy. High photo-thermal conversion has been already reported for metastable 1T MoS_2_ nanosheets and nanoflakes.^13–15,38,39^ The high activity of amorphous MoS_2_ in the Hydrogen Evolution Reaction^32^ interpreted by similar local bonding to that in 1T MoS_2_ (ref. 16) and considering high biocompatibility and stability *etc.* makes amorphous MoS_2_ a promising candidate for a new generation agent in photothermal therapy. The light–heat conversion of the amorphous film in the NIR therapeutic spectral window will be discussed in more detail in Section 4.5.

### 4.3 Transition from an amorphous to a polycrystalline phase

From previous sheet resistance measurements and XPS study, three stages were identified while annealing the films: (i) amorphous as-deposited state and its relaxation, (ii) progressive crystallization, and finally, (iii) grain enlargement and texturization.^6^ Here we supplement our previous findings with an ellipsometry study exploring optical constant evolution along with all these stages. As optical properties are closely related to electrical and structural properties, it is expected that similar trends will be captured in the optical behavior as well.

#### 4.3.1 Relaxation in the amorphous phase

**Fig. 3:**
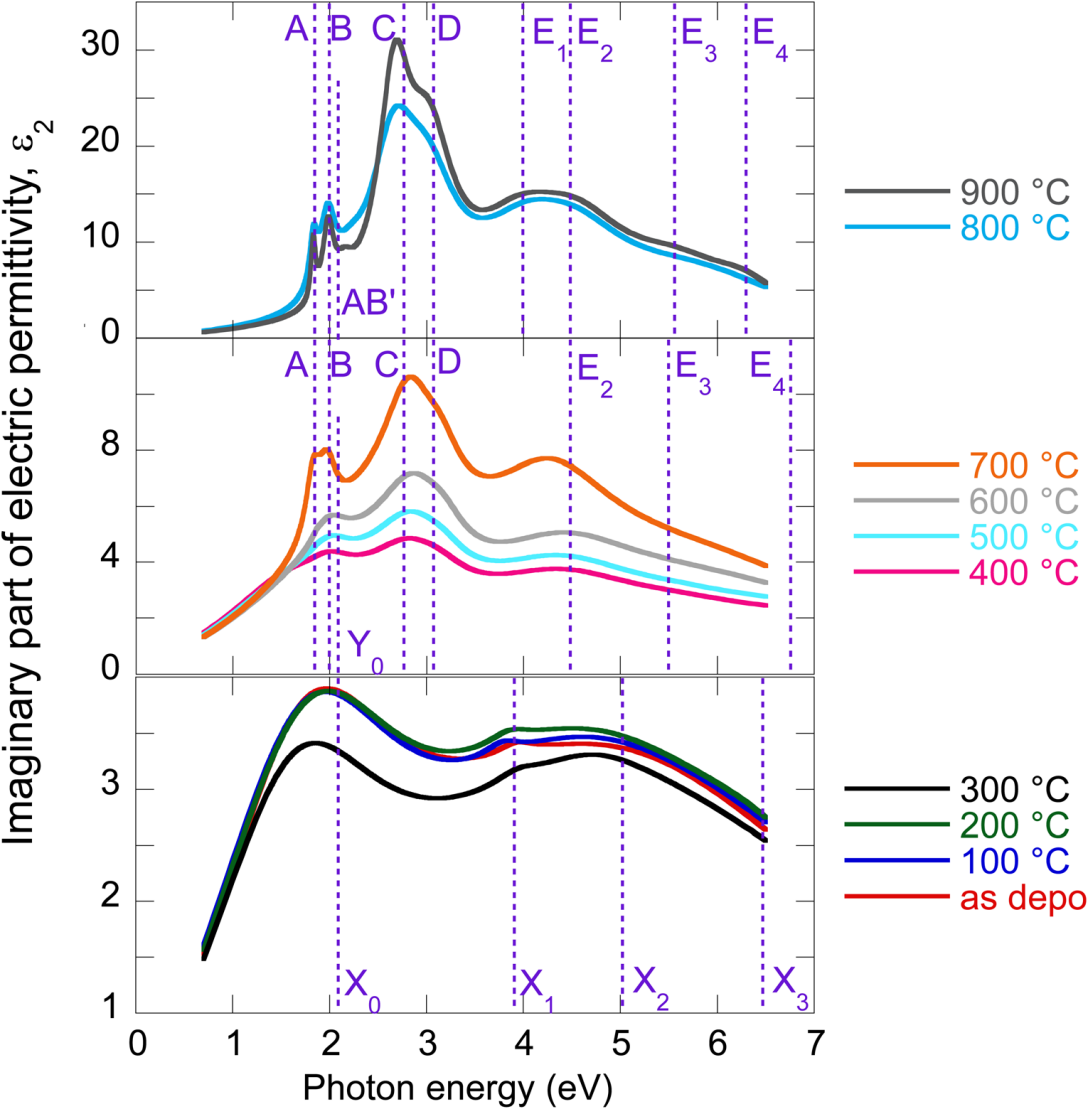
Imaginary part of electric permittivity for as-deposited and annealed MoS_2_ films with an indication of the electronic transitions.

The as-deposited amorphous film and films annealed up to 300 °C present only moderate changes in the optical properties. This is indicated in Fig. 3 in terms of the spectral dependence of imaginary parts of the electric permittivity. The two Lorentz oscillators of 2.1 and 3.9 eV identified for the as-deposited state remain roughly in their positions even for 100 °C, 200 °C, and 300 °C annealed films (*cf.* also Table 1). Additionally, the broad oscillator located for the as-deposited film at 5.9 eV is split into two contributions. Therefore, four total oscillators were sufficient to parameterize the model dielectric function. We assign the slight changes in optical constants to moderate relaxation processes (bounds re-orientation) that stabilize the as-deposited phase by an intake of thermal energy. The transition energy values are listed in Table 1. A moderate change in optical properties in this temperature range is consistent with XRD, XPS, and electric transport measurements.^5,40,41^ Nevertheless, it should be noted that the optical spectra corresponding to 300 °C annealed films are out of trend compared to the other curves, typically falling below what was expected. The reason for this is unclear, but it could be related to slight surface oxidation or specific relaxation processes.

#### 4.3.2 Progressive crystallization of the amorphous phase

When films are further annealed in the temperature range 400–800 °C gradual crystallization takes place. Before we start the discussion of the specific evolution of the film optical constants, it is worth mentioning that during crystallization local bond rearrangement of the 1T′@2H amorphous phase (benefitting from the more energetically stable 2H phase) is generally expected, simultaneously initiated by nucleation of nanocrystalline grains, that would eventually augment in volume with increasing annealing temperature reaching a pure 2H polycrystalline phase (limit case already presented and discussed for the 900 °C annealed film).^42^ All of these structural modifications would influence the film's optical properties. Accordingly, the parameterization of the model dielectric function as the films are annealed at higher temperatures requires a larger number of oscillators (*cf.*Table 1 and Fig. 3). Spectral deconvolution of electric permittivity in a graphical form for all as-deposited and annealed films is provided in the ESI (see Fig. S2).†

The electric permittivity of the 400 °C annealed film contains already contributions of the B, C, and D excitons that are characteristic of the 2H MoS_2_ phase and were discussed in the previous text. From 500 °C, all four main excitons A, B, C, and D become present and gain oscillator strength with increasing annealing temperature. This is consistent with the previously presented picture of preferential 2H bond re-ordering and nucleation of 2H phase crystalline grains. The blue shift of the C exciton resonant energy with respect to the polycrystalline 900 °C film (from 2.67 eV to 2.87 eV, *cf.*Table 1) can be interpreted by the gradual layering of the crystallized 2H phase in nanosized crystalline grains. It is well known that due to the quantum confinement, the excitonic energy is blue-shifted for few layered 2H MoS_2_ and this is most pronounced for C, D, and A excitons (*cf.*Table 1). Referring to literature values,^23^ resonant energy 2.84–2.87 eV, determined in our case for the C exciton (films annealed between 400 and 700 °C), corresponds to 2–5 layered MoS_2_. Further annealing (800 °C and 900 °C annealed films) shifts this energy towards bulk-like values that correspond in our case to augmented crystalline grains with more than 15 S–Mo–S layers, where the C exciton energy is located at 2.67 eV. A similar shift is detected in the case of A and D excitons as well. The spectral position of the B exciton is nearly independent consistently with literature reports.^20,21,28^

*Y*_0_ electronic transition located around 2.1–2.7 eV seems to be of a similar origin (*i.e.*, the contribution of the homopolar Mo–Mo bonds) to the *X*_0_ transition in the as-deposited and relaxed amorphous phases locally 1T, 1T′, and 2H coordinated. On the other hand, the *X*_1_–*X*_3_ transitions of the amorphous phase seem to be replaced (for the films annealed between 400 and 700 °C) with the *E*_2_–*E*_4_ transitions that already capture the spectral position of the pure 2H polycrystalline phase (900 °C annealed film). A slight variation in their values with annealing temperature is due to the presence of other phases with different filling factors.

Although the nature of the bonding and structural changes is rather complex during the film annealing, we have also considered a rough approximation considering the MoS_2_ film as a mixture of an amorphous phase hosting small 2H crystalline inclusions (nucleated grains, increasing in volume with annealing temperature). The optical constants of the polycrystalline film annealed to 800 °C approximated the pure 2H crystalline phase. This choice was due to the expected slight optical anisotropy of the 900 °C annealed film induced by its partial texturing (discussed in more detail later in the text). In other words, the percentage of the crystalline phase at 800 °C was set to 100%. The as-deposited film approximated the amorphous phase. Applying Maxwell-Garnet and Bruggeman's effective medium theories, we were able to estimate an increase of the filling factor of the 2H crystalline phase: 7% (400 °C), 12% (500 °C), 18% (600 °C) and 48% (700 °C). It is worth noting that relatively good fit quality was obtained with a mean squared error (MSE) value of about 6–12 (*cf.*Table 2). The above-mentioned effective medium approximations contain an additional parameter, depolarization factor *Q*, that is related to the shape of inclusions. Adjusting it in a fitting procedure yielded its value to be zero for 400–600 °C annealed films and *Q* = 0.31 for the 700 °C annealed film. This could be related to the needle-like structure of nucleation centers that increase in volume toward the spherical geometry for 700 °C annealing. The increasing volume fraction of the semiconductive 2H phase makes the films less conductive, which is consistent with increased sheet resistance reported in ref. 6.

**Table:** Determined parameters of effective medium approximation. MG and BG stand for Maxwell-Garnett and Bruggeman's EMA, respectively. SR is the surface roughness, *Q* the depolarization factor, and MSE the mean squared error

Temp.	MG/BG	*Q*	Amorph. %	Crystal %	Thickness	SR	MSE
700	BG	0.31	52	48	33	2	6
600	MG	0	82	18	55	0	11
500	MG	0	88	12	60	2	12
400	MG	0	93	7	62	5	12

#### 4.3.3 Preferential grain orientation

MoS_2_ thin films annealed at 800 °C (and also 900 °C) do not present Mo–Mo related *Y*_0_ transition resulting in the loss of metallic character. The only contributions to the electric permittivity here are the A, B, and C excitons, along with transitions in critical points of BZ that can be related to the pure 2H MoS_2_ phase. Well-developed and resolved excitonic and CP-related spectral features prove the high quality of 2H MoS_2_ films. Even more pronounced excitonic peaks in the case of the 900 °C annealed film could be related to crystal grain augmentation and improvement of overall crystalline quality. The texturing or preferential orientation of the atomic layer, as proved by XRD diffractograms, is difficult to support by ellipsometry due to limited access to the out-of-plane optical response, as already discussed earlier. The presence of excited states of A and B excitons (oscillator located at about 2.2 eV) is another complementary proof of the high quality of the 800 °C and 900 °C annealed MoS_2_ films with a well developed crystalline 2H phase.

### 4.4 Reflectivity

**Fig. 4:**
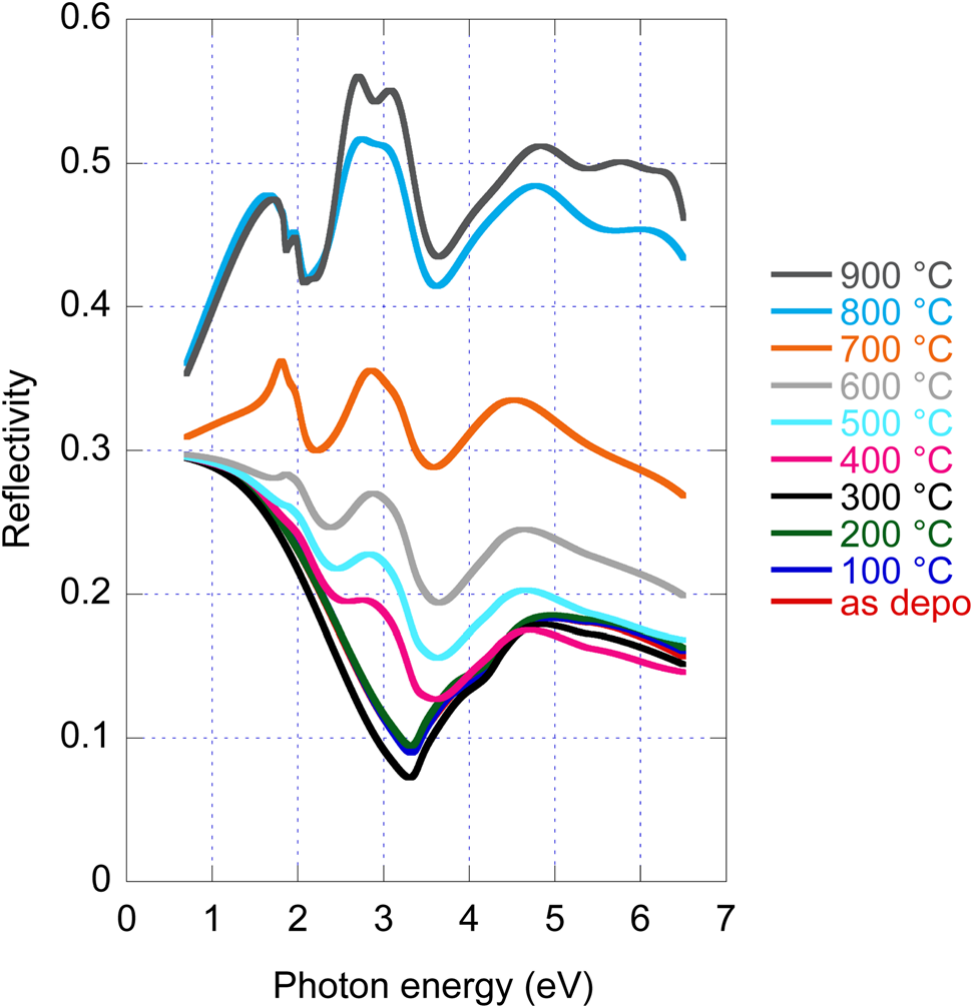
As deposited and annealed MoS_2_ film reflectance calculated for normal incidence and film with a nominal thickness of 50 nm.

Disclosed significant variations of real and imaginary parts of optical constants along amorphous to crystalline transition, both stable phases, opens a new potential path for the application of this material employing the phase change functionality. For this reason, we provide in Fig. 4 reflectance spectra recorded for all samples with given annealing temperatures. Even if experimental data of *R* are available (angle of incidence 18°), we provide in Fig. 4 re-calculated normal incidence *R* spectra in the entire spectral range. The large increase in reflectance from the amorphous (20%) to crystalline (50%) phase is noticeable.

### 4.5 Light–heat conversion in the NIR therapeutic window

**Fig. 5:**
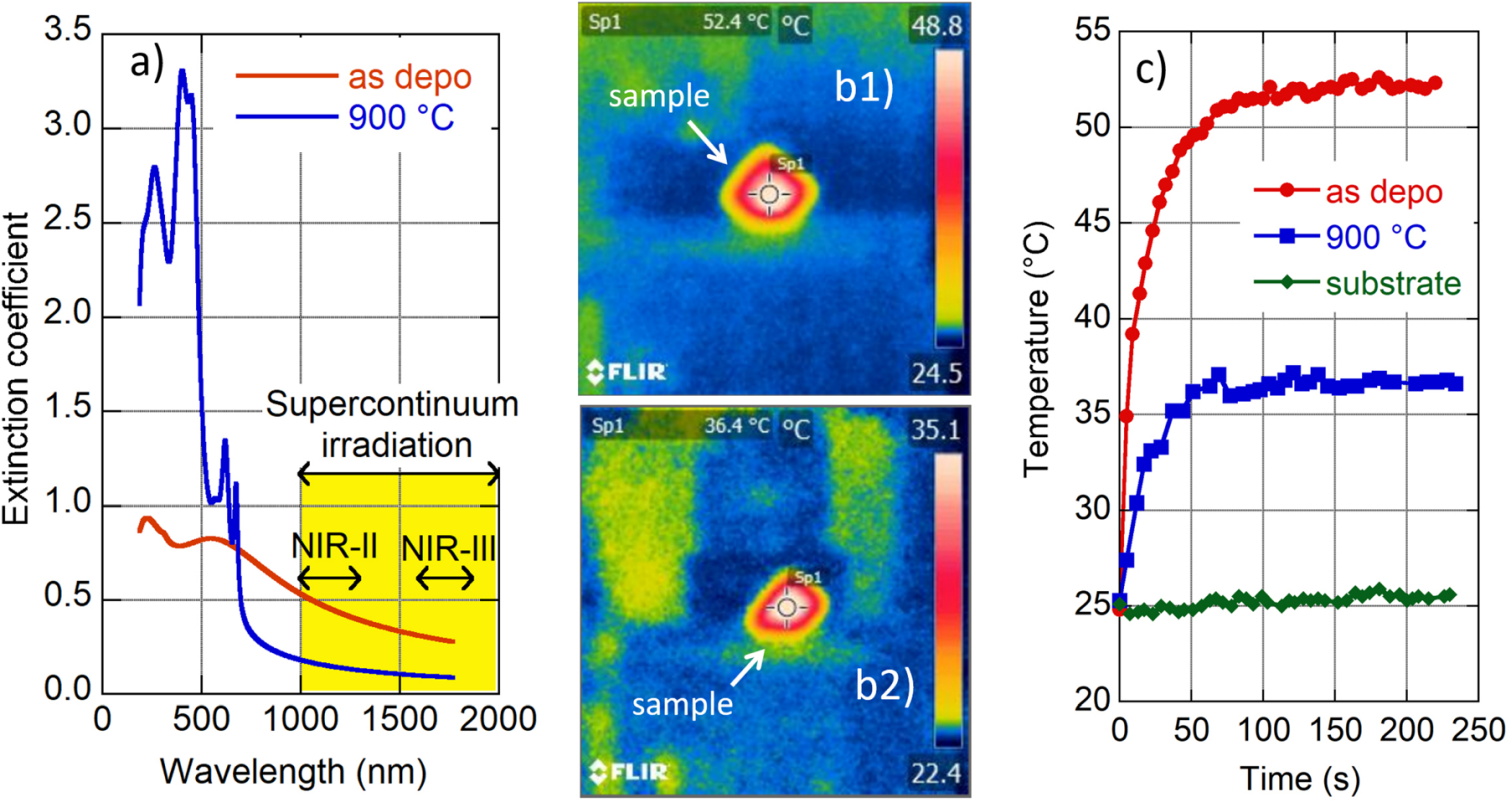
Light–heat conversion in the NIR therapeutic window. Wavelength dependence of the extinction coefficient of as-deposited (red line) and 900 °C annealed (blue line) films with an indication of spectral ranges of applied supercontinuum radiation and biological transparency windows (NIR-II and NIR-III) (a). Photos from the infrared camera of as-deposited (b1) and annealed (b2) samples for saturated temperature. Time evolution of recorded temperature for the naked substrate (green diamonds), as-deposited (red circles) and 900 °C annealed (blue squares) films (c).

We carried out a supplementary experiment to support our conviction that amorphous MoS_2_ is a promising candidate for photothermal therapy. Amorphous (as deposited) and crystalline (900 °C annealed) films were exposed to light from a supercontinuum laser source. A long pass filter was inserted into the incident beam to restrict its spectral range in the interval 1000–2200 nm and total transmitted power of 340 mW. The selected spectral range covers the following parts of the biological transparency window:^15^ NIR-II (1000–1350 nm) and NIR-III (1600–1870 nm) as indicated in Fig. 5a. We are interested in light–heat conversion exclusively generated in the film; therefore we selected samples with transparent fused silica substrates. An infrared camera was used to record film temperature increase while the samples were irradiated (*cf.* images in Fig. 5b(1) and b(2)). The observed time evolution of sample temperature is plotted in Fig. 5c.

The naked substrate was considered as well as a control sample. Irradiation did not cause any temperature change in this case as expected due to fused silica transparency in the NIR. On the other hand, irradiation of the amorphous and 2H polycrystalline MoS_2_ films resulted in a steep increase of recorded temperature shortly after the beam was switched on (during the first minute), and then the temperature was gradually saturated (*cf.*Fig. 5c). The saturation temperature is significantly higher for amorphous (≈52 °C) than the crystalline (≈36 °C) film, which is consistent with the higher values of the amorphous MoS_2_ extinction coefficient with respect to its 2H crystalline phase (see Fig. 5a). Zhou and coworkers reported a temperature increase of polyvinylpyrrolidone-modified MoS_2_ nanodots to a value of about 32 °C (2H phase) and to about 53 °C (1T phase) when irradiated in a solution (50 ppm) with a NIR laser (1064 nm, 1.0 W cm^−2^).^14^ Additionally, Yougbaré *et al.* reported an increase of temperature in 1T phase MoS_2_ nanosheet solution (100 μg mL^−1^) to about 54 °C when irradiated with an 808 nm laser diode with a power density of 1.0 W cm^−2^.^43^ Although the mentioned results are not directly comparable with our findings, due to different experimental arrangements (light source spectral range and its power, a form (nanoparticle solution and thin film) of MoS_2_), our data show that light–heat conversion in the amorphous MoS_2_ film displays a similar enhanced tendency to that reported for MoS_2_ in its 1T crystalline phase. Moreover, as the amorphous phase is thermodynamically more stable than 1T, we suggest that amorphous MoS_2_ is an even better candidate for the photothermal agent for photothermal therapy.

## 5 Conclusions

A spectroscopic ellipsometry study of as-deposited magnetron sputtered MoS_2_ thin films disclosed the original UV-VIS-NIR optical properties of the amorphous MoS_2_ phase with a mixed 1T′@2H local order. The subsequent gradual annealing of the films and their systematic optical investigation revealed a large optical contrast along the transition from the amorphous to the 2H MoS_2_ crystalline phase that is attractive for phase-change applications. The evolution of the obtained optical constants was correlated with material structural modifications capturing the nucleation of few layer nanostructured 2H centers and their enlargement toward the polycrystalline phase. The results of light–heat conversion in the NIR therapeutic window show so far uncovered potential of amorphous MoS_2_ as an agent for photothermal therapy. Applied spectroscopic ellipsometry proved to be a sensitive and reliable tool providing consistent results with other characterization tools such as XRD, XPS, and sheet resistance measurements.

## Author contributions

J. Mistrik: methodology, investigation – ellipsometry and reflectance spectra recording, together with light–heat conversion measurements, formal analyses of the data, their validation and curation, and writing – original draft. M. Krbal: supervision, conceptualization, and writing – review & editing. V. Prokop: sample preparation. J. Prikryl: investigation.

## Conflicts of interest

There are no conflicts to declare.

## Supplementary Material

NA-005-D3NA00111C-s001
