# Supplementary material for: Giant change of MoS2 optical properties along amorphous–crystalline transition: broadband spectroscopic study including the NIR therapeutic window
Source: Nanoscale Adv. 2023 Apr 18;5(11):2911–20. doi: 10.1039/d3na00111c (PMC10228343; doi:10.1039/d3na00111c)
Supplement: NA-005-D3NA00111C-s001 [file NA-005-D3NA00111C-s001.pdf]

Electronic Supplementary Material (ESI) for Nanoscale Advances.
This journal is © The Royal Society of Chemistry 2023

Electronic supplementary information (ESI)

Giant change of MoS_2 optical properties along amorphous-crystalline transition: broadband spectroscopic study including NIR therapeutic window

J. Mistrik,[ab] M. Krbal,[a] V. Prokop[a] and J. Prikryl[a]

[a]*Center of Materials and Nanotechnologies, Faculty of Chemical-technology, University of Pardubice, Studentska 95, 53210 Pardubice, Czech Republic*

[b]*Institute of Applied Physics and Mathematics, Faculty of Chemical-technology, University of Pardubice, Studentska 95, 53210 Pardubice, Czech Republic*

Contents

S1 Absorbance measurements of MoS_2 films on transparent substrates **S2**

S2 Spectral deconvolution of MoS_2 electric permittivity **S3**

S3 Wavelength dependence of MoS_2 refractive index and extinction coefficient **S4**

Number of Supportimg Information Pages: 4

Number of Supportimg Information Figures: 3

Number of Supportimg Information Tables: 0

S1 Absorbance measurements of MoS_2 films on transparent substrates

Optical transmittance spectra of as-deposited and annealed MoS_2 films were recorded in the wavelength range from 200 to 800 nm with the step of 2 nm using a JASCO V-570 UV/VIS/NIR spectrophotometer. The transparent substrates of fused silica were used for the transmittance measurements. Fig. S1 shows corresponding results presented in the form of absorbance. Experimental spectra of ellipsometry parameters, nearly-normal reflectance, and transmittance were treated simultaneously by multiple sample method. The right side of Fig. S1 indicates a change of the film color along the amorphous to 2H MoS_2 crystalline transition.

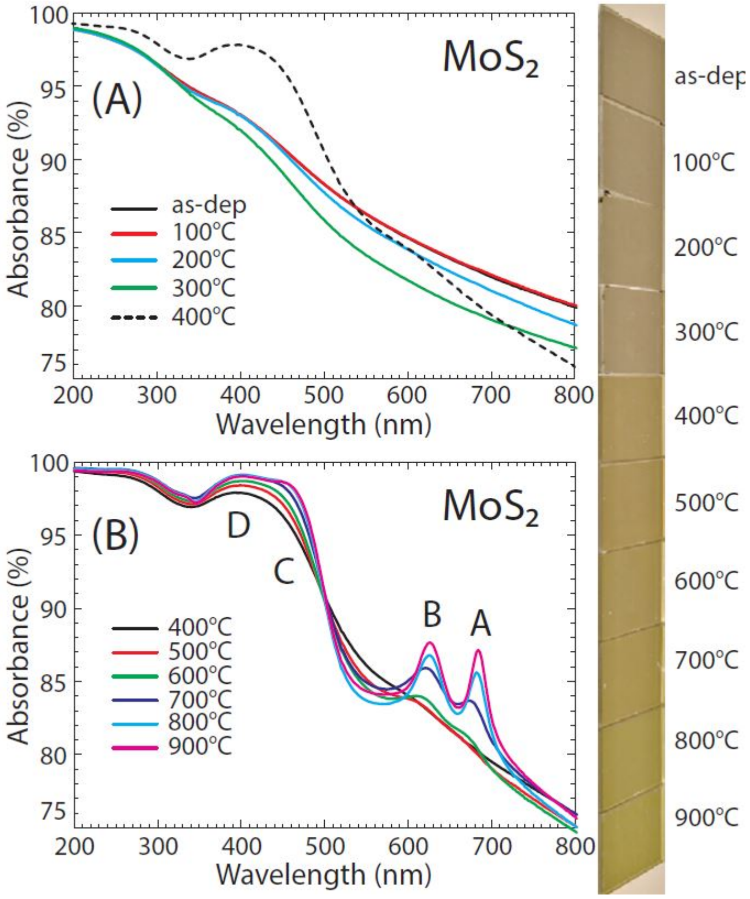

Figure S1 Absorbance spectra of MoS_2 thin films deposited on transparent fused quartz substrates. The excitonic features A, B, C, and D are also indicated.

S2 Spectral deconvolution of MoS_2 electric permittivity

MoS_2 electric permittivity was parameterized as a sum of Lorentz oscillators. The resulting spectra of as-deposited and annealed films are plotted in Fig. S2 together with the individual contributions.

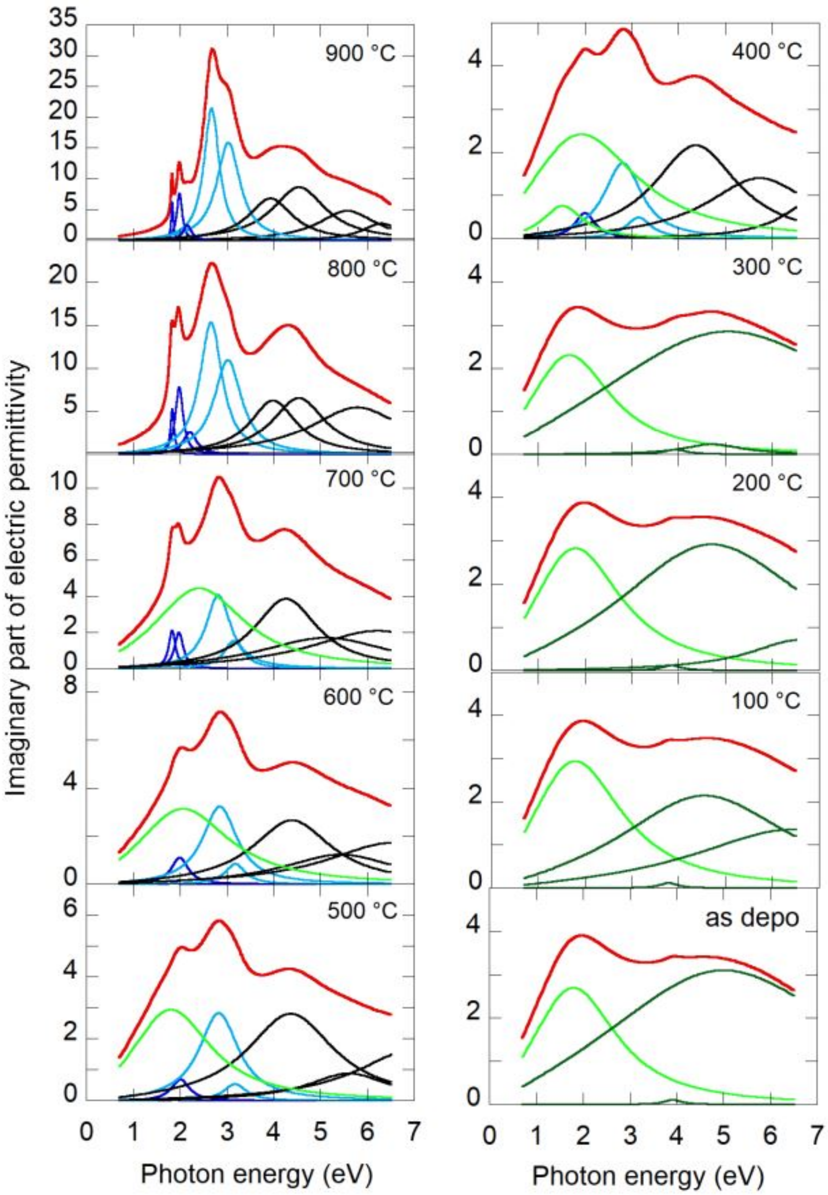

Figure S2 Spectral deconvolution of MoS_2 electric permittivity along the amorphous to 2H MoS_2 crystalline transition. Presented are the imaginary parts and their particular contributions.

S3 Wavelength dependence of MoS_2 refractive index and extinction coefficient

Optical constants of MoS_2 are often reported in the literature in the form of the wavelength dependence of complex refractive index. For this reason, we provide in Fig S3 spectra of refractive index and extinction coefficients recalculated from the electric permittivity.

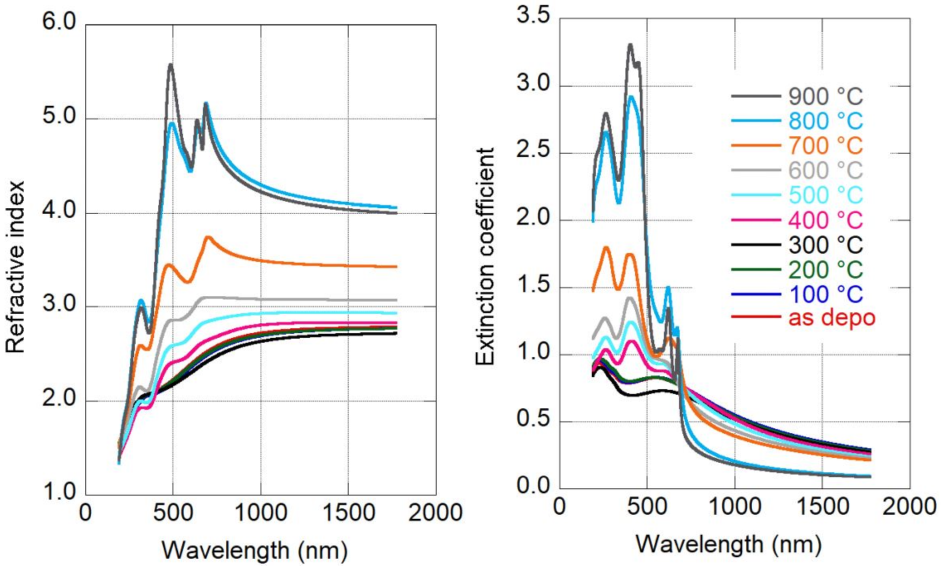

Figure S3 Spectral dependence of refractive index and extinction coefficient of MoS_2 of as-deposited and annealed films.
